# Capillary-Mediated Vitrification: Preservation of mRNA at Elevated Temperatures

**DOI:** 10.1208/s12248-022-00723-z

**Published:** 2022-06-16

**Authors:** Sankar Renu, Mary Shank-Retzlaff, Jenny Sharpe, Laura Bronsart, Pravansu Mohanty

**Affiliations:** Upkara, Inc, 1600 Huron Parkway Bldg 520, Rm 2390, Ann Arbor, Michigan 48109 USA

**Keywords:** biopreservation, lyophilization, mRNA, stability, transfection

## Abstract

RNA is a fundamental tool for molecular and cellular biology research. The recent COVID-19 pandemic has proved it is also invaluable in vaccine development. However, the need for cold storage to maintain RNA integrity and the practical and economic burden associated with cold chain logistics highlight the need for new and improved preservation methods. We recently showed the use of capillary-mediated vitrification (CMV), as a tool for stabilizing temperature-sensitive enzymes. Here, we demonstrate the use of CMV as a method to preserve mRNA. The CMV process was performed by formulating a green fluorescent protein (GFP)-encoding mRNA with common excipients, applying the solution to a porous support, referred to as the scaffold, and drying the samples under vacuum for 30 min. The CMV preserved samples were stored at 55 °C for up to 100 days or 25 °C for 60 days and analyzed by electrophoresis and for transfection efficiency in a cell-based assay. The 55 °C-stressed mRNA exhibited comparable electrophoresis banding patterns and band intensity when compared to a frozen, liquid control. Additionally, the CMV stabilized mRNA maintained 97.5 ± 8.7% transfection efficiency after 77 days and 78.4 ± 3.9% after 100 days when stored 55 °C and analyzed using a cell-based assay in the CHO-K1 cell line. In contrast, a liquid control exhibited no bands on the electrophoresis gel and lost all transfection activity after being stored overnight at 55 °C. Likewise, after 60 days at 25 °C, the CMV-processed samples had full transfection activity while the activity of the liquid control was reduced to 40.1 ± 4.6%. In conclusion, CMV is a simple formulation method that significantly enhances the thermal stability of mRNA, requires minimal processing time, and could enable formulation of mRNA that can tolerate exposure to temperatures well above 25 °C during shipment and deployment in extreme environments.

**Graphical abstract:**

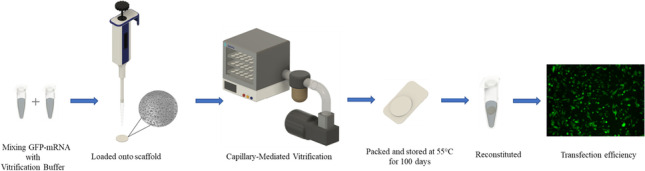

## INTRODUCTION

RNA is a key tool for genomic research and is widely used in human and veterinary diagnostic testing and, more recently, in therapeutic treatments (1, 2). The FDA has approved three small interfering RNA (siRNA)–based drugs for orphan diseases and rare genetic disorders (1, 3). In addition, two mRNA-based vaccines have been approved to use for COVID-19 (4).

Despite the expanded use of RNA in both analytical and clinical applications, RNA can be challenging to work with due to its inherent instability and numerous degradation pathways. RNA-based materials typically require storage at − 20 or − 80 °C, or under liquid nitrogen (5). RNA samples are generally transported on dry ice resulting in higher shipment costs, increased shipping weights, logistical complexity, and stringent regulations (5). Long-term storage of RNA samples requires construction of biorepositories with extensive cold storage, and the infrastructure required to monitor and maintain the refrigeration units (6). These issues suggest a critical need for alternative preservation processes that would enable ambient storage of RNA.

We recently reported a novel biopreservation process called capillary-mediated vitrification (CMV) (7). CMV leverages the naturally-occurring process of capillary evaporation to rapidly remove moisture from an aqueous matrix without freezing or boiling, enabling transition of the biological molecules into a stable glassy state (7, 8). Unlike traditional lyophilization processes, the CMV process does not require a freezing step and the process can be completed in less than 1 h. Using the CMV process, we showed that firefly luciferase, a temperature-sensitive enzyme that is routinely stored frozen at − 80 °C, can be preserved and maintain its full activity when stored at 25 °C for up to 42 days. The CMV-stabilized luciferase showed > 70% activity at temperatures up to 45 °C for 42 days and > 50% activity after 1 week at 55 °C (8).

Here, we demonstrate the use of CMV to stabilize a green fluorescent protein-encoding mRNA (GFP-mRNA). GFP-mRNA encodes a 26.6 kDa fluorescent protein, with 5′ Cap 1 structure and 3′ poly(A) tail. GFP-encoding mRNA is widely used to study transfection and expression of mammalian cells. In the present study, GFP-mRNA was preserved, stored at 55 °C and 25 °C, and monitored using electrophoresis and a cell-based assay for transfection efficiency. These results suggests that it may be possible to formulate mRNA in a manner that will enable ambient shipping and deployment even in environments where temperatures routinely exceed 25ºC.

## MATERIALS AND METHODS

### Preparation of CMV Scaffold and Vitrification Buffer

The CMV process was performed as previously described (7, 8). Briefly, the scaffold (Cat#1010108001, Upkara, Ann Arbor, MI) material was manually cut into 8-mm-diameter circles. Samples were prepared as described below using Upkara Samadhi® buffer (Cat#1012005001, Upkara, Ann Arbor, MI), referred to as vitrification buffer (VB).

### GFP-mRNA and Lipofectamine

Dasher GFP-mRNA was purchased from Aldevron, WI, aliquoted and stored at − 80 °C as per the recommendations. Lipofectamine MessengerMAX transfection reagent was obtained from Thermo Fisher Scientific, CA, stored at 4 °C.

### Sample Preparation and CMV Process

To prepare the vitrified samples, equal volume of GFP-mRNA (1 mg/mL) and VB were mixed and 6 µl of formulated solution applied to the scaffold. The loaded scaffolds were placed into the vitrification chamber and dried for 30 min. During the vacuum cycle, the shelves were held at a constant temperature. The vitrification chamber consisted of a custom-built vacuum chamber containing temperature-controlled shelves (Cat#1014001001, Upkara, Ann Arbor, MI). After drying, the vitrified samples were sealed in Mylar pouches (Impak, CA). The packaged vitrified samples, and liquid controls containing the required concentration and volume, were stored at 55 °C for up to 100 days or 25 °C for 60 days in a temperature-controlled incubator. Two independent sample preparations were used for each time point.

### Agarose Gel Electrophoresis

At the scheduled time points, vitrified samples were eluted using FluoroBrite Dulbecco’s Modified Eagle medium (DMEM) (Thermo Fisher Scientific, CA) with gently vortexing and a 10-min incubation at the room temperature. The eluted mRNA (125 ng) was loaded and run on a 1.2% agarose gel, stained using SYBR™ Green II RNA gel stain (Thermo Fisher Scientific, CA), visualized under Gel Doc EZ System (BioRad, CA), the images were captured, and the percentage of band intensity calculated with respect to a control consisting of mRNA stored frozen samples using the Image Lab 6.0.1 software (BioRad, CA). A Millennium™ RNA marker size range from 0.5 to 9 kb was used as a ladder.

### Cell Culture and GFP-mRNA Transfection

Chinese hamster ovary subclone (CHO-K1) cells were cultured in FluoroBrite DMEM supplemented with 10% fetal bovine serum (FBS), GlutaMAX, and antibiotics, incubated at 37 °C with 95% air and 5% CO_2_. After confluent, 0.2 × 10^6^ cells/well were seeded on Corning® 96-well flat-bottom black polystyrene tissue culture plates (Sigma, MO) using FluoroBrite DMEM and incubated overnight at 37 °C. For each time point, three replicate samples (vitrified or liquid) each containing 250 ng frozen GFP-mRNA, and stored at 55 °C and 25 °C were mixed with 1 μL Lipofectamine using FBS free FluoroBrite DMEM and transfected. After 24-h incubation, the transfected cells were imaged using the GFP filter set in a Leica Microsystems fluorescence microscope (Leica Microsystems Inc, IL) at 20 × magnification (scale: 100 μM). The total fluorescence intensity was measured by processing the 2 to 4 images using ImageJ software (NIH, MD) and the percentage of transfection efficiency with respect to the frozen stored mRNA control calculated.

## RESULTS

### CMV Process

**Fig. 1:**
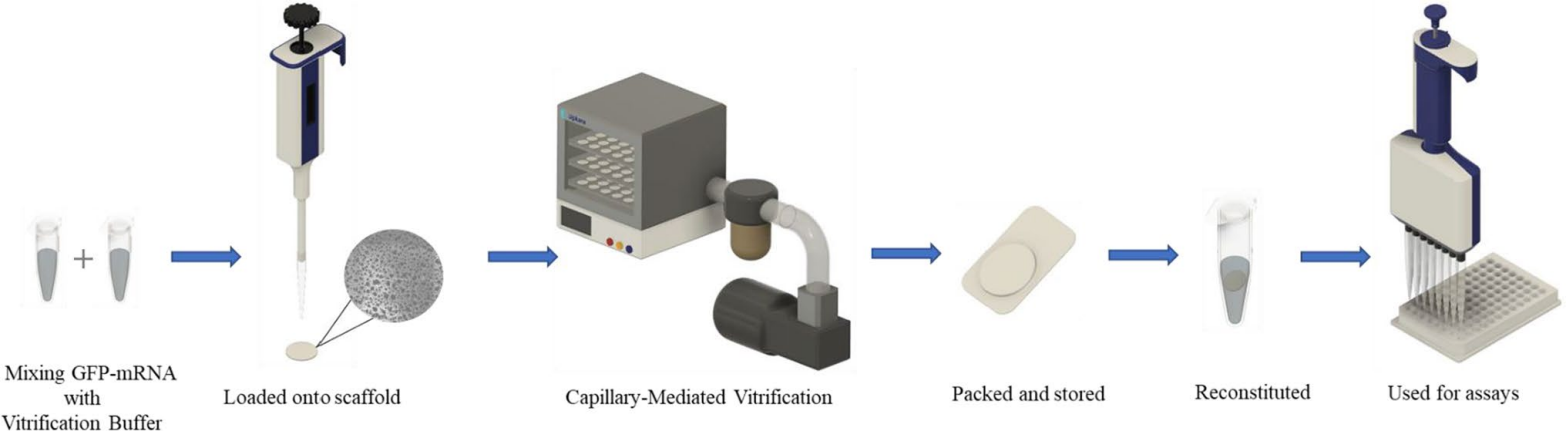
Schematic illustration of overall capillary-mediated vitrification process

We used a biocompatible, porous scaffold as the solid base for the vitrification process. As described previously (8), the pores in the scaffold provide an increased surface area and enable capillary evaporation, accelerating drying and preventing boiling when dried under vacuum. The VB provides the necessary excipients to ensure the formation of an amorphous solid and prevents nucleation, which can lead to crystallization. A schematic illustration of the overall CMV process is shown in Fig. 1. Scaffold morphology in the pre- and post-CMV process, temperature profile and drying cycle time optimization, product residual moisture, and amorphous nature were described previously (8).

### CMV Processed GFP-mRNA Thermostability

**Fig. 2:**
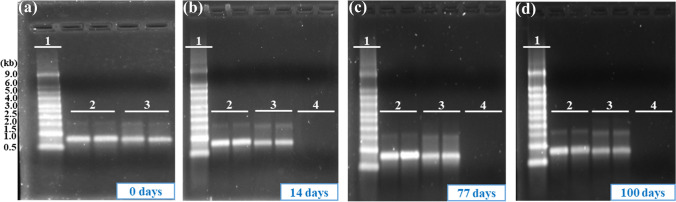
GFP-mRNA thermostability enhanced by CMV process. The vitrified and liquid control samples were stressed at 55 °C or stored frozen and analyzed by gel electrophoresis and visualized using Gel Doc. The electrophoresis results for samples stored at 55 °C for **a** 0 day; **b** 14 days; **c** 77 days; and **d** 100 days. 1, RNA ladder; 2, frozen control samples stored at − 80 °C; 3, CMV-processed samples stored at 55 °C; and 4, liquid control samples stored at 55 °C. Two independent samples were used for each time point. (GFP-mRNA, green fluorescent protein-encoding mRNA, CMV, capillary-mediated vitrification)

The vitrified mRNA was analyzed by electrophoresis prior to being exposed to the stress conditions and the results compared to a frozen liquid control. All of the samples exhibited similar band patterns and signal intensity compared to the frozen, liquid control sample (Fig. 2a). This result indicates that the CMV process itself does not adversely impact the quality of the mRNA.

To evaluate the thermostability of the CMV-processed RNA, we subsequently stressed the vitrified samples and liquid controls at 55 °C. The samples were tested after 14, 77, and 100 days at 55 °C and compared to a frozen liquid control (Fig. 2b–d). At each time point, the CMV samples and frozen controls exhibited comparable band patterns. The CMV stabilized samples exhibited slightly lower intensity at all the tested timepoints but no low molecular species (bands below the main RNA band) were observed (Table I). The liquid mRNA sample was completely degraded after 14 days at 55 °C (Fig. 2).

**Table I:** Percentage of Transfection Efficiency and Band Intensity of CMV Processed and 55 °C for 100 days Stressed Samples. Two Independent samples were used to Calculate the Percentage of Transfection Efficiency and Band Intensity Respective to Frozen Control. Values are Represented as the mean ± SD

Days	CMV processed samples stored at 55 °C	
	Transfection efficiency (%)	Agarose gel band intensity (%)
0	107.9 ± 4.9	71.5 ± 4.9
14	103.0 ± 3.3	62.0 ± 4.2
77	97.5 ± 8.7	76.0 ± 4.2
100	78.4 ± 3.9	66.0 ± 1.4

**Fig. 3:**
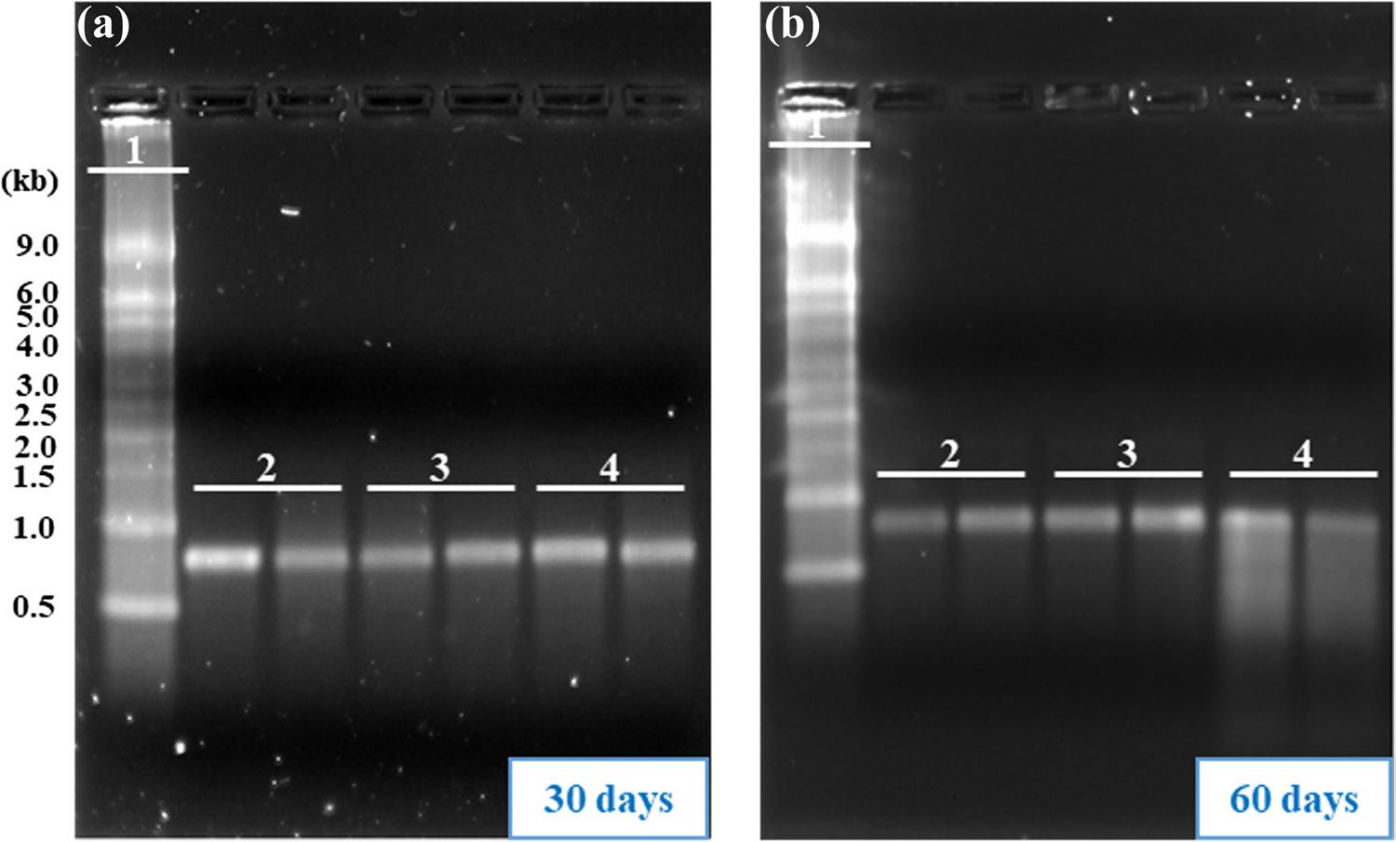
CMV process maintains mRNA integrity long term at ambient temperature. The vitrified and liquid control samples were stored at 25 °C or frozen GFP-mRNA run on the agarose gel and visualized using Gel Doc. The electrophoresis results for samples stored at 25 °C for **a** 30 days and **b** 60 days. 1, RNA ladder; 2, frozen control samples stored at − 80 °C; 3, CMV-processed samples stored at 25 °C; and 4, liquid control samples stored at 25 °C. Two independent samples were used for each time point. (GFP-mRNA, green fluorescent protein-encoding mRNA, CMV, capillary-mediated vitrification)

Based on the results obtained at 55 °C, a second stability study was initiated at 25 °C for the purpose of to evaluate the feasibility of storing CMV-stabilized RNA under ambient conditions. After either 30 or 60 days at 25 °C storage, the CMV processed samples had similar band intensity compared to the frozen control samples (Fig. 3). The band intensity observed for the liquid control samples stored at 25 °C was 98.0 ± 7.1% and 58.5 ± 26.2% at 30 and 60 days, respectively (Table II).

**Table II:** Percentage of Transfection Efficiency and Band Intensity for Vitrified and Control Samples Stored at Room Temperature for 30 and 60 Days. Two Independent Samples, were used to Calculate the Percentage of Transfection Efficiency and Band Intensity Corresponding to Frozen Control. Values are Represented as the mean ± SD

Days	CMV processed samples stored at 25 °C		Liquid control samples stored at 25 °C	
	Transfection efficiency (%)	Agarose gel band intensity (%)	Transfection efficiency (%)	Agarose gel band intensity (%)
30	95.5 ± 0.2	97.0 ± 32.5	58.1 ± 10.8	98.0 ± 7.1
60	142.3 ± 32.2	119.5 ± 23.3	40.1 ± 4.6	58.5 ± 26.2

### Long-term Transfection Activity of Preserved GFP-mRNA

**Fig. 4:**
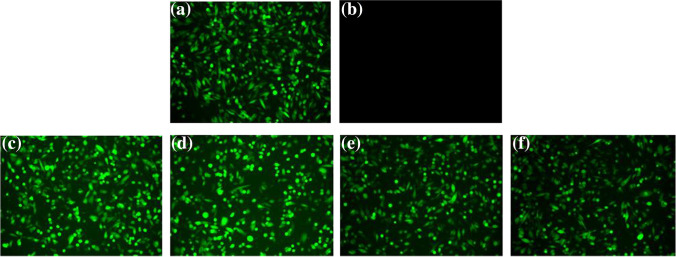
CMV-processed GFP-mRNA maintained comparable transfection activity of frozen control samples after stressed at long term. The vitrified and liquid control samples stressed at 55 °C or frozen-stored GFP-mRNA were transfected on CHO-K1 cells using Lipofectamine. The transfection efficiency of **a** frozen control samples stored at − 80 °C; **b** liquid control samples stored overnight at 55 °C; CMV-processed samples stored at 55 °C for **c** 0 days; **d** 14 days; **e** 77 days; and **f** 100 days. Three replicates of two independent samples were used for each time point. (GFP-mRNA, green fluorescent protein encoding mRNA; CMV, capillary-mediated vitrification)

**Fig. 5:**
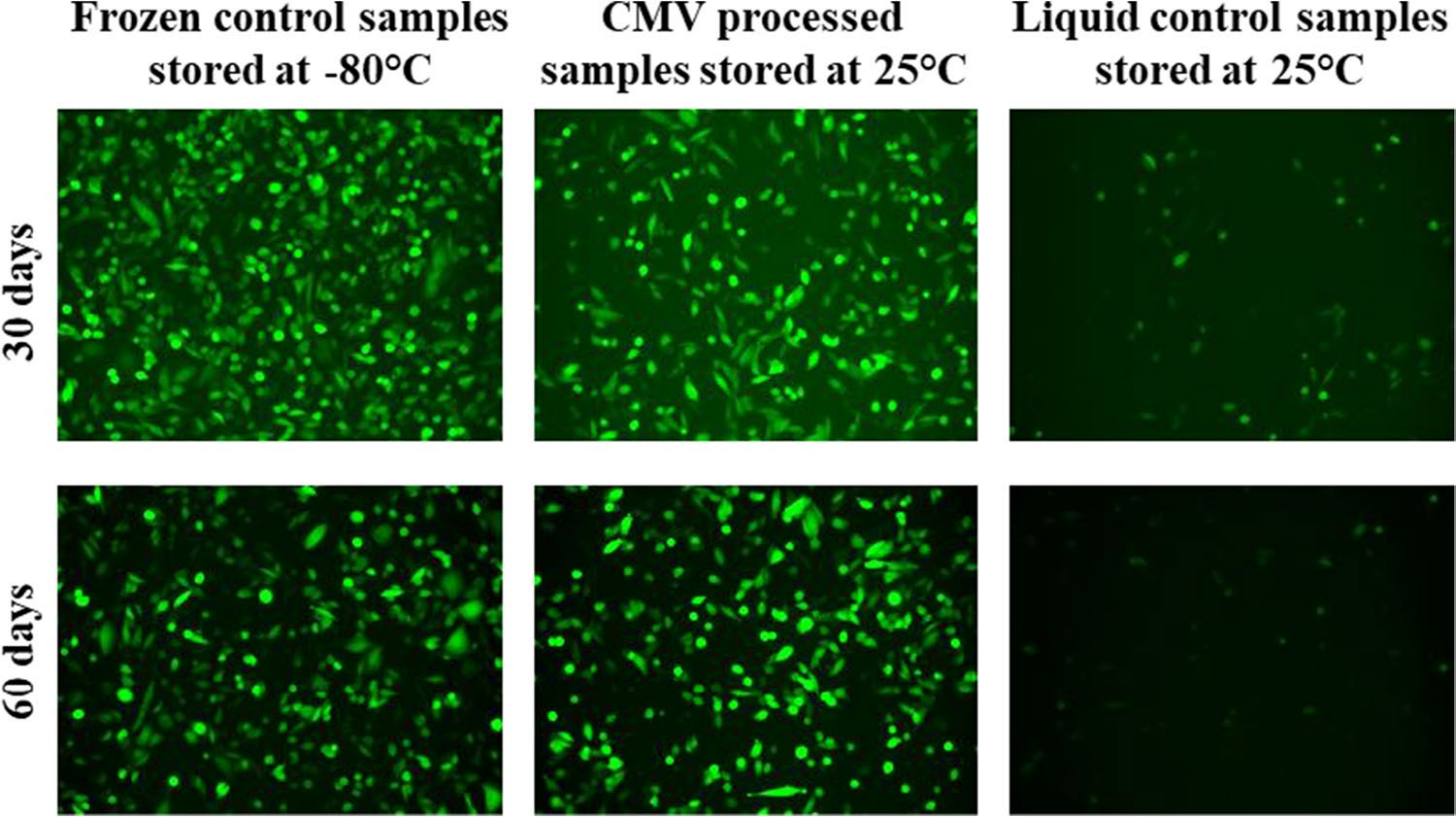
Transfection activity of CMV-processed GFP-mRNA stored at room temperature. The vitrified and liquid control samples stored at 25 °C for 30 and 60 days or frozen-stored GFP-mRNA were transfected on CHO-K1 cells using Lipofectamine and the images captured. Three replicates of two independent samples were used for each time point. (GFP-mRNA, green fluorescent protein-encoding mRNA, CMV, capillary-mediated vitrification)

To examine the functionality of preserved and stressed samples, we transfected GFP-mRNA using CHO-K1 cells and Lipofectamine as a transfection agent. Unsurprisingly, cells transfected with the frozen, liquid control sample emitted high fluorescence signals after 24 h of transfection (Fig. 4a). Remarkably, the unstressed and 55 °C-stressed CMV samples emitted comparable fluorescence signals to the frozen control at each of the stability time points (0, 14, 77, and 100 days) (Fig. 4c–f). Total transfection efficiency of the preserved samples was 97.5 ± 8.7% on 77 days and 78.4 ± 3.9% for 100 days compared to the frozen, liquid control (Table I). Consistent with the electrophoresis analysis, the liquid control showed no transfection activity after overnight storage at 55 °C (Fig. 4b). The CMV processed samples stored at 25 °C for 30 and 60 days exhibited 95.5 ± 0.2% and 142.3 ± 32.2% of the transfection efficiency was observed relative to the frozen control, respectively, whereas the transfection efficiency for the liquid control stored at 25 °C was 58.1 ± 10.8% after 30 days and 40.1 ± 4.6% after 60 days (Fig. 5 and Table II).

## DISCUSSION

RNA is a critical component for both molecular and cellular biology research. Because of its inherent instability, RNA is generally stored frozen to preserve activity and to avoid degradation. However, even when stored frozen at ultracold temperatures (− 80 °C and below), the RNA may not maintain full activity when stored for extended periods. The limited shelf-life and complex logistics associated with cold-storage have a significant negative impact on both the financial costs and manpower required to execute laboratory studies with RNA. Likewise, RNA-based vaccines and therapeutics also suffer from these issues. One critical example is the impact of cold storage requirements on the availability of COVID-19 vaccines in low-income countries. Some estimates suggest that as a result of the shorter shelf-life and limited availability of cold storage in the developing world, as much as half of the mRNA vaccines produced to date have been wasted (4). The RNA markers degradation during long-term storage of forensic and clinical samples are also unsolved issues (9, 10).

Additionally, ultra-low-temperature freezers used to store RNAs cost up to $20,000/year to run continuously and each freezer releases up to 35,000 pounds of carbon dioxide (11). Freeze-drying or lyophilization can mitigate some of the issues with frozen storage, but often requires product-specific optimization, is both capital- and energy-intensive, requires long drying cycle time, and may not be suitable for complex formulations including those containing complex lipid assemblies (12–14). The storage of RNA in RNAshell minicapsules has been demonstrated as an alternative to lyophilization (5, 15). However, these processes cannot be easily adapted for therapeutic applications, and it is unclear whether the stabilization solution may interfere with some analytical applications including both cell-based assays and *in vivo* potency testing of drugs and vaccines. Development of RNA formulations that are stable under ambient conditions, suitable for use in *in vivo* and therapeutic applications, and can be utilized in regions where the temperatures routinely exceed 25 °C and are tolerant of temperature excursions that can occur during shipping and storage are needed. Compared to existing RNA stabilization methods, the CMV process is highly flexible and is compatible with *in vivo* applications. The scaffold can be designed to fit into a variety of containers ranging from standard lyophilization vials, microcentrifuge tubes, and disposable plastic ware or can be incorporated into novel containers that are process and work-flow specific. Additionally, the CMV process leverages only common excipients that are considered safe for both human health products and animal studies, while simultaneously minimizing the risk of analytical matrix interference.

In this study, we used the novel CMV process to preserve GFP-mRNA. CMV leverages a porous scaffold to increase surface area and enables capillary evaporation, enabling a rapid drying cycle without the risk of boiling or the need for a freezing step (7). CMV-preserved GFP-mRNA maintained both its integrity and transfection activity when stored long-term at 25 °C and higher temperature. The CMV processed samples had comparable transfection efficiency when stored at either room temperature or 55 °C, respectively, compared to the protein expression (fluorescence signal) observed for a frozen mRNA control (16, 17).

Although further stability studies are required, this data suggests CMV process may enable long-term storage of mRNA at the room or above temperatures and could provide an opportunity to reduce the cold chain requirements for mRNA vaccines. However, the impact of the CMV process on the structure of the lipid nanoparticle (LNP) formulation must be fully characterized to ensure that there is no change to the LNP size, shape, and product potency.

This proof-of-concept study also demonstrates the potential for using CMV to simplify the storage and transportation of analytical and clinical samples. As an example, the CMV process could be used to enhance the stability of neonatal blood spots which although they are stored dried, currently require long-term, frozen storage. The CMV process may also allow samples collected at international clinical study sites to be shipped under ambient conditions to a central testing laboratory for processing.

The CMV technology is expected to be applicable to a wide range of biological molecules. Previous studies have shown that a similar process, including the same excipient mixture and vacuum cycle, can also be used to stabilize proteins and enzymes (18–20). Thus, the process is believed to be easily adaptable to different types of biomolecules (7) and may enable storage, distribution, and deployment of RNA-based reagents, clinical samples, and therapeutics in regions where the cold chain is not readily available.
